# Synthesis and applications of high-performance P-chiral phosphine ligands

**DOI:** 10.2183/pjab.97.026

**Published:** 2021-11-11

**Authors:** Tsuneo IMAMOTO

**Affiliations:** *1 Graduate School of Science, Chiba University, Chiba, Japan.

**Keywords:** P-chiral phosphine ligand, phosphine–borane, catalytic asymmetric synthesis, asymmetric hydrogenation, enantioselection mechanism

## Abstract

Metal-catalyzed asymmetric synthesis is one of the most important methods for the economical and environmentally benign production of useful optically active compounds. The success of the asymmetric transformations is significantly dependent on the structure and electronic properties of the chiral ligands coordinating to the center metals, and hence the development of highly efficient ligands, especially chiral phosphine ligands, has long been an important research subject in this field. This review article describes the synthesis and applications of P-chiral phosphine ligands possessing chiral centers at the phosphorus atoms. Rationally designed P-chiral phosphine ligands are synthesized by the use of phosphine–boranes as the intermediates. Conformationally rigid and electron-rich P-chiral phosphine ligands exhibit excellent enantioselectivity and high catalytic activity in various transition-metal-catalyzed asymmetric reactions. Recent mechanistic studies of rhodium-catalyzed asymmetric hydrogenation are also described.

## 1 Introduction

Catalytic asymmetric synthesis is one of the most efficient methods for the production of optically active compounds used in pharmaceuticals, agrochemicals, fragrances, and so on.^1–5)^ Among available methodologies for asymmetric catalysis, transition-metal-catalyzed reactions have been studied for more than six decades, and a variety of reactions of this class have been developed. The enantioselectivities and catalytic activities of the reactions are generally largely affected by the chiral ligands as well as the center metals.

Among various types of chiral ligands, phosphine ligands play an outstanding role in asymmetric catalysis owing to their intrinsic property of coordinating strongly to the transition metals and to promoting the catalytic reactions. A great number of chiral phosphine ligands have been synthesized and used in this class of asymmetric reactions.^6–20)^

**Figure 1.**
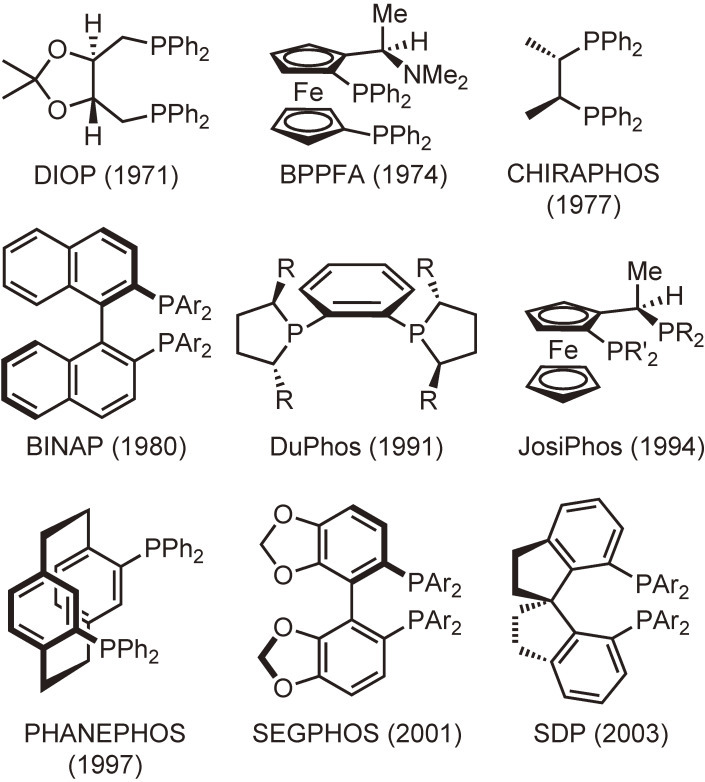
Representative examples of bisphosphine ligands with backbone chirality. Figures in parentheses are the years when the ligands were published in journals.

Chiral phosphine ligands are divided into two classes. One class includes backbone chirality ligands that possess their stereogenic centers on the linking carbon chain. Representative examples of this class are shown in Fig. 1. Most of the hitherto reported chiral phosphine ligands belong to this class, and some of them are used as benchmark ligands not only for the synthesis of various chiral compounds but also for the development of new catalytic asymmetric reactions.

**Figure 2.**
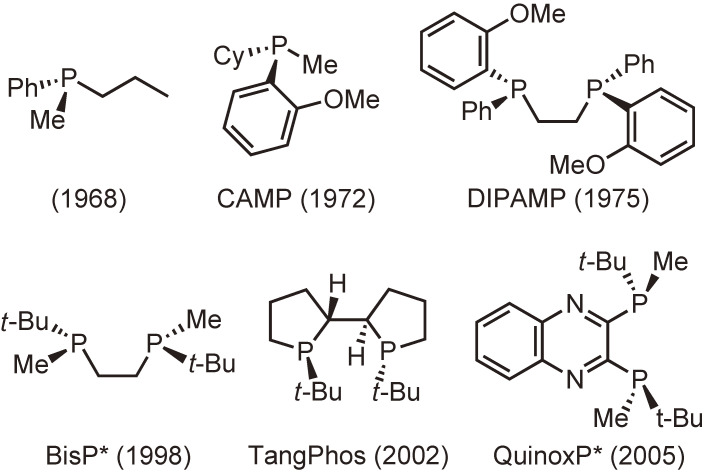
Representative examples of P-chiral phosphine ligands.

Another class is composed of P-chiral (P-chirogenic or P-stereogenic) phosphine ligands possessing their stereogenic centers at the phosphorus atoms. Figure 2 shows the representative examples of this class. Although P-chiral phosphine ligands are small in number compared with backbone chirality ligands, some of the ligands play a critical role in the early stage of the study on Rh-catalyzed homogeneous asymmetric hydrogenation.^21–23)^ In particular, the use of DIPAMP ligand, which was developed by Knowles and coworkers at Monsanto Company, provided high enantioselectivities of up to 96% in 1975, the highest at that time.^24–26)^ The same ligand was successfully employed in the manufacture of (*S*)-3,4-dihydroxyphenylalanine (l-DOPA) that is used to treat Parkinson’s disease.^27,28)^ However, despite those landmark achievements, P-chiral phosphine ligands including DIPAMP have not been widely used for more than 20 years. This is mainly attributed to the synthetic difficulties in generating optically pure P-chiral phosphines. In addition, the fact that some phosphines bearing electron-withdrawing groups are stereochemically unstable and gradually racemize via pyramidal inversion even at room temperature may have discouraged researchers. Another reason is that many backbone chirality phosphine ligands such as BINAP have been synthesized and their superior catalytic performance has been proven in various asymmetric syntheses.

On the other hand, in our studies of the synthesis and reactions of phosphine–boranes, we found that P-chiral phosphine ligands could be synthesized via the stereospecific removal of the BH_3_ group of chiral phosphine–boranes. This finding inspired us to study the design and synthesis of new P-chiral phosphine ligands and their applications in catalytic asymmetric synthesis. This review article contains our studies on phosphine–boranes and P-chiral phosphine ligands. It also mentions how we discovered new research themes and developed them, taking on the many challenges that came with the work and overcoming the difficulties along the way.

## 2 Prologue to the study of P-chiral phosphine ligands

### 2.1 Use of cerium element in organic synthesis.

After receiving my Ph.D. degree in physical organic chemistry in 1972, I went on to work at four universities to pursue further studies in organic synthesis. For 8 years, I worked as a postdoctoral fellow or an assistant professor, seeking tenure. Fortunately, in 1980, I was appointed assistant professor at Chiba University. I was 37, and that appointment gave me an excellent opportunity to begin my own research work. However, at that time, my research group was quite small, and it was almost impossible to undertake a big project. I decided to seek out new research subjects in the largely unexplored areas. Perusing the periodic table of elements in an effort to find elements that had not yet been utilized in organic synthesis, I arrived at the conclusion that lanthanide elements were the perfect choice. Although Professors Kagan and Luche had already reported their pioneering and outstanding investigations at that time,^29–32)^ I believed that this area offered the exciting possibility of discovering new and useful synthetic methods. Among 15 lanthanides, I focused on cerium element because cerium exists in high natural abundance, and its major salts are commercially available at affordable prices.

**Scheme 1.**
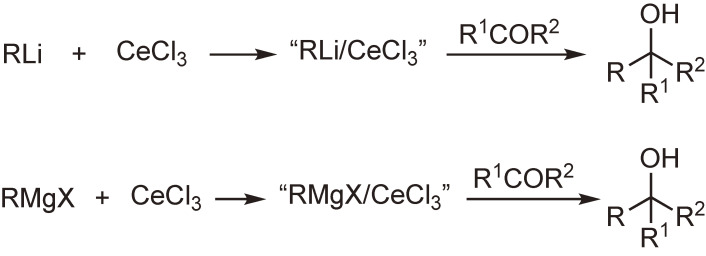
Preparation of organocerium reagents and their reactions with carbonyl compounds.

**Scheme 2.**
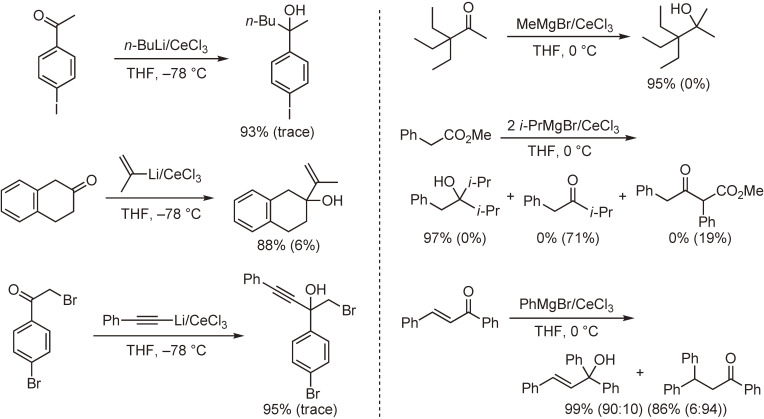
Representative examples of the reactions of organocerium reagents with carbonyl compounds. Values in parentheses indicate the yields obtained in the reactions without the use of cerium chloride.

After many attempts using metallic cerium or cerium(III) salts, we succeeded in preparing new reagents called “organocerium reagents” or “cerium(III)-modified organometallic reagents”. These reagents are readily prepared by the reaction of organolithiums or Grignard reagents with anhydrous cerium(III) chloride in tetrahydrofuran. The cerium reagents undergo nucleophilic addition reactions with carbonyl groups more efficiently than the parent organometallic reagents. Thus, the reagents react with various carbonyl compounds to afford the corresponding normal addition products in high yields, even though the substrates are susceptible to so-called abnormal reactions, such as enolization, reduction, conjugate addition, condensation, and metal–halogen exchange reaction (Scheme 1).^33–35)^ Several reaction examples are shown in Scheme 2. This method, together with Knochel’s improved method,^36,37)^ is widely used in organic synthesis.

### 2.2 Working with phosphine–boranes.

**Scheme 3.**
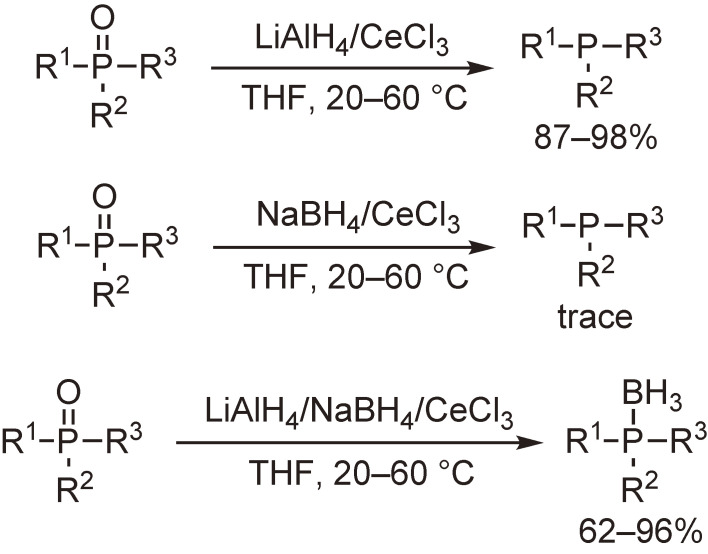
Transformation of phosphine oxides into phosphines or phosphine–boranes.

The above-mentioned cerium chloride methodology was extended to a LiAlH_4_/CeCl_3_ system to examine the enhanced or modified reducing ability of LiAlH_4_. Our initial experiments on the reduction of phosphine oxides and organic halides including fluorides demonstrated the powerful reducing ability of LiAlH_4_ under mild conditions.^38)^ Notable is that various phosphine oxides including sterically congested ones were readily converted into the corresponding phosphines in high yields. In contrast, the use of NaBH_4_ instead of LiAlH_4_ afforded only trace amounts of the products. Taking one step further, we conducted the reaction with a three-component reagent, LiAlH_4_–NaBH_4_–CeCl_3_, to find that phosphine–boranes were produced in good to high yields (Scheme 3).^39)^

We were surprised to find that phosphine–borane compounds, including secondary ones having a P–H bond, were virtually inert to air and hardly decomposed even on contact with an acid or a base. Phosphine–boranes possess formal charges of +1 and −1 on the phosphorus and boron atoms, respectively, and are a kind of phosphorus ylide and boron ate complex. I was fascinated by these structural features and decided to study phosphine–boranes from the perspective of organic synthesis. I emphasized the significance of this research to my collaborator students by mentioning that two Nobel Prize Laureates, G. Wittig and H.C. Brown, were involved in our phosphine–borane research. The following studies were conducted in parallel with the synthesis and application of P-chiral phosphine ligands. The main purpose of these studies was to create interesting and fundamentally important chemical species and to find unprecedented reactions by utilizing the characteristic properties of phosphine–boranes.

#### 2.2.1 Generation and reactions of tricoordinate boron dianions.

**Scheme 4.**
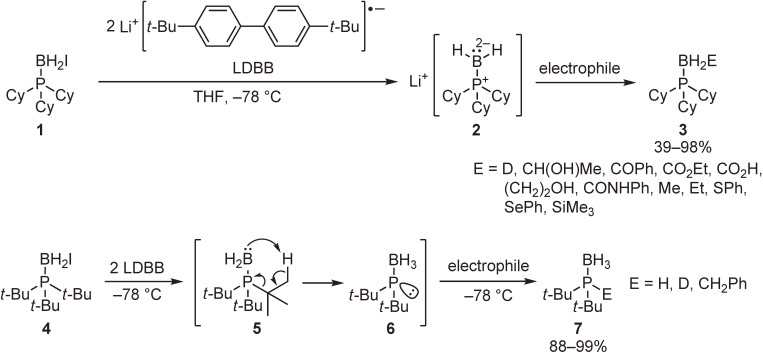
Generation and reactions of tricoordinate boron dianions.

Tricoordinate boron dianions have an isoelectronic relationship with tricoordinate carbanions, which are one of the most important chemical species in organic chemistry. We were interested in whether or not the boron dianions exhibit reactivities similar to the carbanions and intended to generate such chemical species by using phosphine–boranes. After various trials, we succeeded in the generation of the desired chemical species possessing a formal −2 charge on the boron atom, and demonstrated their high nucleophilicity and strong basicity, which are similar to those of carbanions. Thus, tricyclohexylphosphine–monoiodoborane (**1**) was reacted with lithium 4,4′-di-*tert*-butylbiphenylide (LDBB) to generate boron dianion **2**, which was allowed to react with various electrophiles to give the boron-functionalized phosphine–boranes (**3**) (Scheme 4).^40)^ Similarly, the reduction of tri-*tert*-butylphosphine–monoiodoborane (**4**) with LDBB, followed by the reaction with electrophiles, furnished compounds **7**. It is reasonable to consider that the generated boron dianion **5** underwent a pericyclic reaction at −78 ℃ and the resulting phosphide anion **6** was trapped by the electrophiles. This reaction was compared with the corresponding carbanion species, tri-*tert*-butylphosphonium methylide, which was reported to undergo the same type of pericyclic reaction at a much higher temperature (20 ℃).^41)^ These results clearly indicate that the basicity of the boron dianion is greater than that of the corresponding carbanion.

#### 2.2.2 Synthesis of enantiopure tetracoordinate boron compound and stereochemistry of substitution reactions at boron atom.

**Scheme 5.**
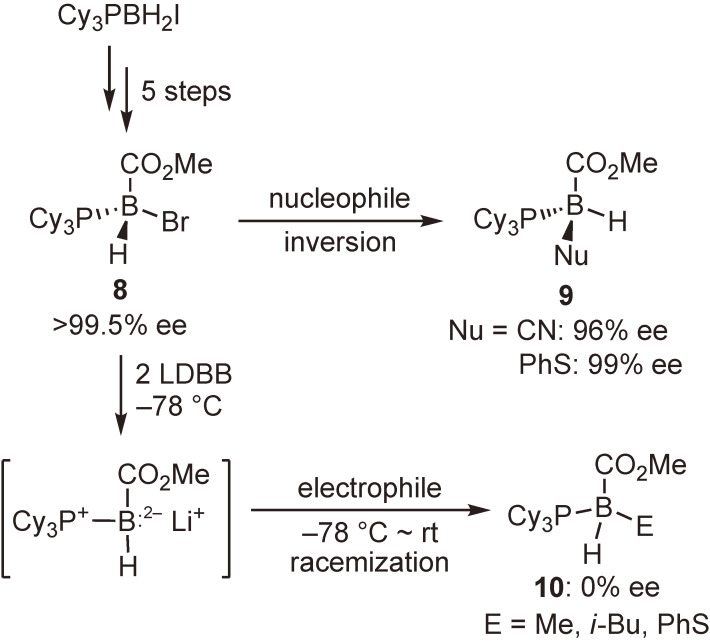
Synthesis of enantiopure tetracoordinate boron compound and substitution reactions at boron atom.

Reports of nucleophilic substitution reactions at the tetracoordinate boron atom abound, but little attention has been given to the stereochemistry of the reactions. We initially succeeded in synthesizing enantiopure B-stereogenic tetracoordinate boron compound **8** containing a bromo substituent as the leaving group and examined the stereochemistry of both the nucleophilic and the electrophilic substitution reactions (Scheme 5).^42)^

The reactions of **8** with nucleophiles afforded the substitution products **9** with excellent enantiomeric excesses. These results apparently demonstrate that the S_N_2 reaction at the sp^3^ boron atom proceeded with inversion of configuration. On the other hand, the reduction of **8** with LDBB, followed by the reactions with electrophiles, provided completely racemized products **10**. These experimental facts can be explained by assuming that the intermediate boranyl radical or the boron dianion species would be stereochemically unstable and undergo rapid stereomutation under the reaction conditions.

#### 2.2.3 Preparation and reactions of boranophosphorylation reagents.

**Scheme 6.**
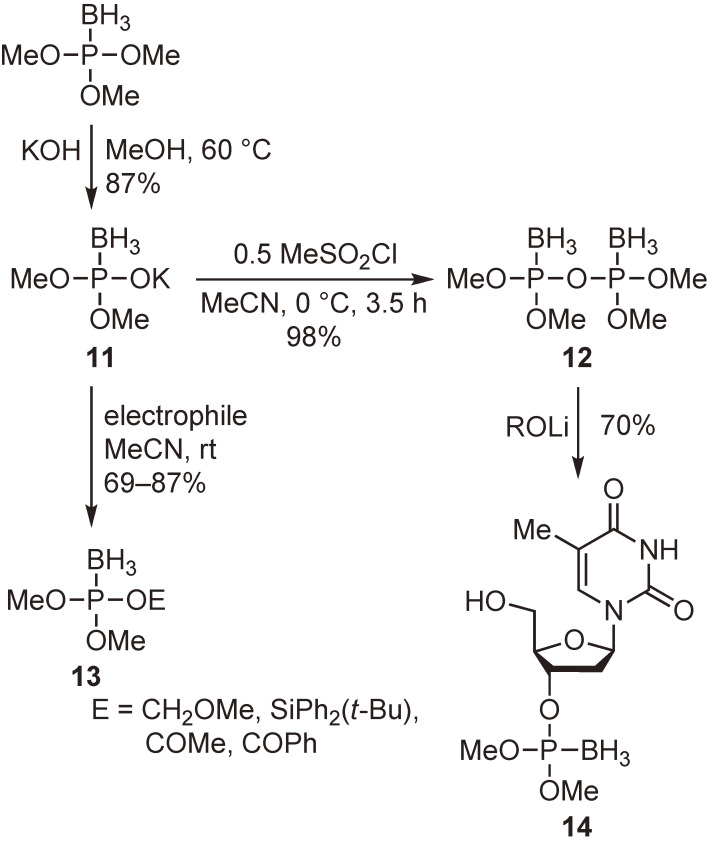
Preparation and reactions of boranophosphorylation reagents **11** and **12**.

Boranophosphates, which have an isoelectronic relationship with phosphates, are useful in biochemical investigations. They also have potential utility as carriers of ^10^B in boron neutron capture therapy for cancer treatment. We tried to develop new reagents for the synthesis of similar compounds. As shown in Scheme 6, dimethyl boranophosphate monopotassium salt **11** and tetramethyl boranopyrophosphate **12** were prepared from the borane adduct of trimethylphosphite. The former compound underwent substitution reactions with various electrophilic reagents to generate compounds **13**, whereas the latter reacted with metal alkoxides to give the corresponding boranophosphate derivatives such as compound **14**.^43)^ These are simple synthetic routes to various boranophosphate derivatives, including the borano analogs of naturally occurring phosphates.

#### 2.2.4 Synthesis and reactions of various phosphine–borane derivatives.

**Scheme 7.**
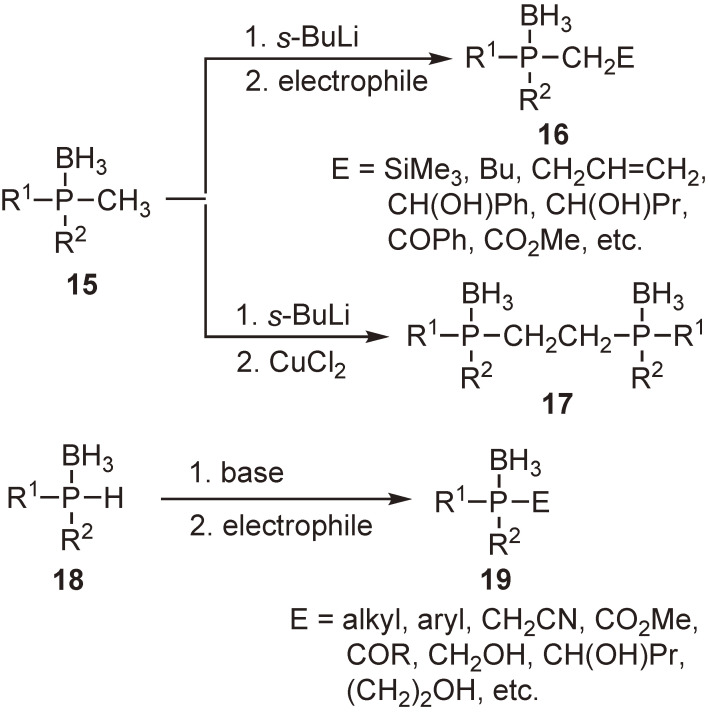
Synthesis of phosphine–borane derivatives from tertiary or secondary phosphine–boranes.

We examined the functionalization of the phosphine moiety of phosphine–boranes. As shown in Scheme 7, tertiary phosphine–boranes **15** bearing a methyl group at the phosphorus atom were subjected to deprotonation, followed by the reaction with alkyl halides or carbonyl compounds to give various phosphine–borane derivatives **16**. Oxidative dimerization proceeded smoothly to afford compounds **17** with the boranato group intact. Secondary phosphine–boranes **18** reacted with a variety of electrophiles in the presence of a base to yield corresponding phosphine–borane derivatives **19**, similarly to the reactions of secondary phosphine oxides.^39,44)^

**Scheme 8.**
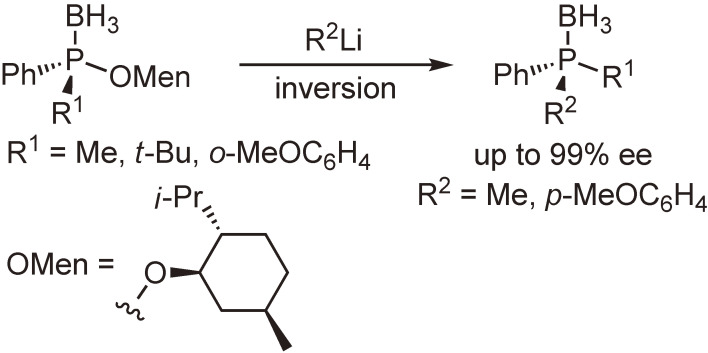
Nucleophilic substitution reactions of enantiopure phosphine–boranes bearing *l*-menthoxy group.^45,46)^

**Scheme 9.**
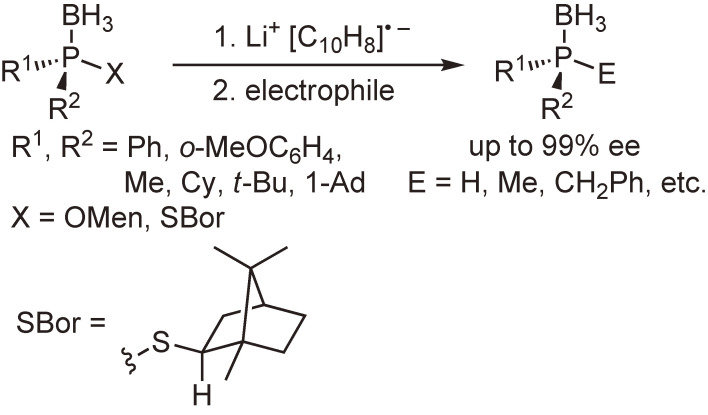
Stereospecific reduction of menthoxyphosphine–boranes or bornylthiophosphine–boranes with lithium naththalenide.^47–49)^

**Scheme 10.**
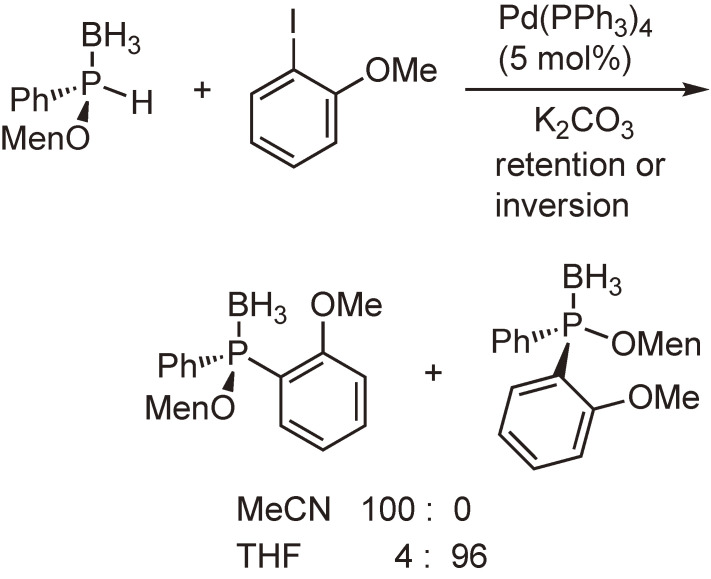
Palladium-catalyzed arylation of enantiopure secondary phosphine–borane.^50,51)^

Our subsequent interest was the synthesis and reactions of optically active phosphine–boranes. Several phosphine–boranes bearing *l*-menthoxy or bornylthio group were prepared in diastereomerically pure form, and their reactions with nucleophiles or one-electron reducing agents were examined. The results shown in Schemes 8–10 indicate that chiral phosphine–boranes can be obtained in excellent enantiopurity, most of which are limited to compounds bearing aryl groups at the phosphorus atom.^45–51)^

**Scheme 11.**
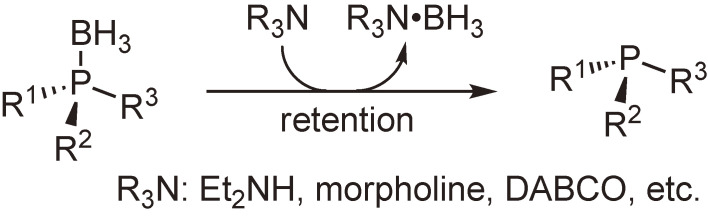
Stereospecific deboranation of phosphine–boranes to form optically active phosphines.

Our study of the reactivities of phosphine–boranes was extended to the transformation of the boranato group moiety. After many trials, we found that phosphine–boranes reacted with amines such as diethylamine and 1,4-diazabicyclo[2.2.2]octane to produce parent tertiary phosphines and amine–boranes. Further trials using optically active phosphine–boranes indicated that this deboranation proceeded with complete retention of configuration (Scheme 11).^39,44)^ We were delighted with the finding of this simple and unprecedented reaction and were convinced that various phosphines including optically active ones can be synthesized via this deboranation process. It is noteworthy that the BH_3_ group plays the role of protecting group of the labile phosphine moieties.

**Scheme 12.**
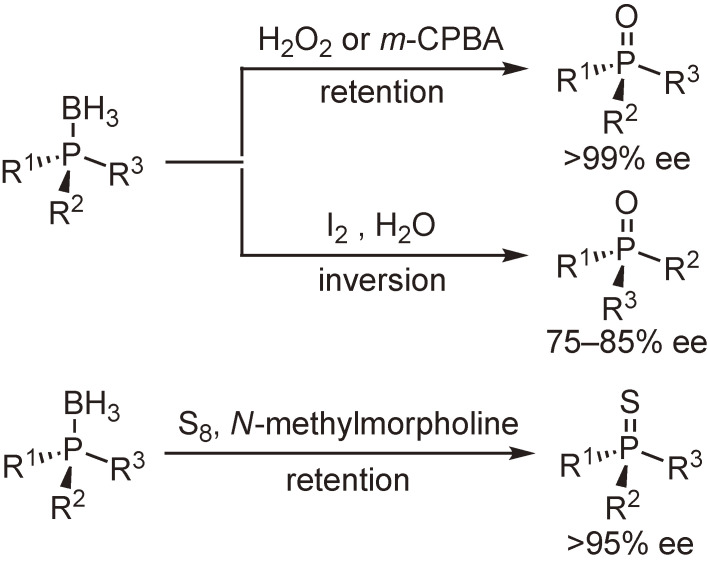
Stereospecific conversion of phosphine–boranes into phosphine oxides or phosphine sulfides.

The conversion of the boranato group of phosphine–boranes into oxygen or sulfur atom was examined. The reaction with hydrogen peroxide or *m*-chloroperbenzoic acid proceeded at 0 ℃ with complete retention of configuration to provide the corresponding phosphine oxides. On the contrary, the reaction with iodine in the presence of water occurred at room temperature with inversion of configuration, while it was accompanied by partial racemization. The reaction with sulfur in the presence of *N*-methylmorpholine proceeded at 110 ℃ while retaining stereochemical integrity to give the corresponding phosphine sulfides in almost quantitative yields (Scheme 12).^52)^

**Scheme 13.**
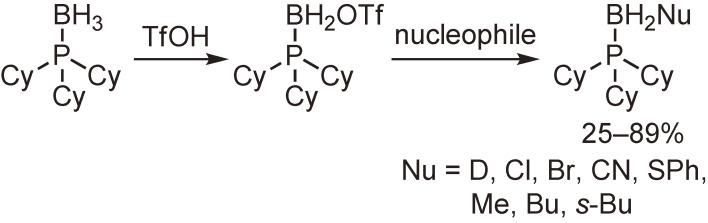
Synthesis and substitution reactions of the trifluoromethanesulfonyloxy derivative of tricyclohexylphosphine–borane.

**Scheme 14.**
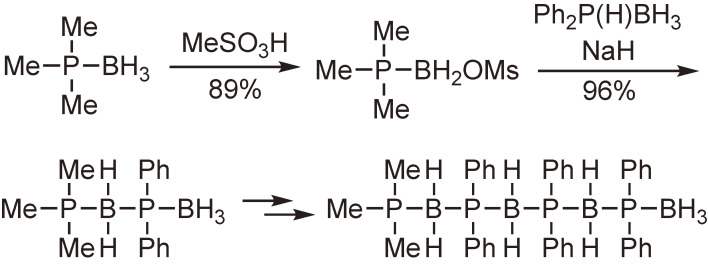
Synthesis of organophosphorus compounds with a linear P–B bond chain.

The functionalization of phosphine–boranes at the boron atom was then examined. It was found that one of the hydrogen atoms of the boranato group was readily replaced with a trifluoromethanesulfonyloxy or methanesulfonyloxy group by the reaction with trifluoromethanesulfonic acid or methanesulfonic acid. The resulting triflate and mesylate were subjected to a nucleophilic substitution reaction to afford the functionalized phosphine–boranes (Schemes 13 and 14).^53,54)^

**Scheme 15.**
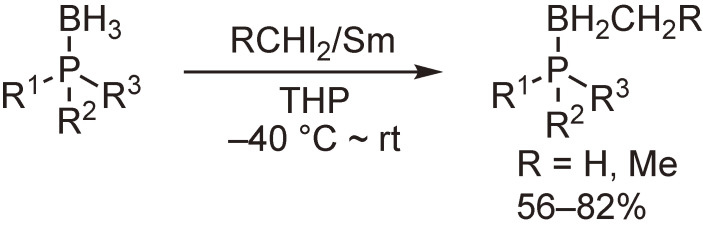
Methylene insertion reactions of samarium carbenoids into the B–H bond of phosphine–boranes.

Direct boron–carbon bond formation was also examined through the reactions with metal carbenoids. Scheme 15 shows our observation that samarium carbenoids undergo methylene insertion into the B–H bond to afford B-alkylated compounds.^55)^

## 3 Synthesis of P-chiral phosphine ligands

### 3.1 Synthesis of P-chiral bisphosphine ligands with aryl groups at phosphorus atoms.

**Scheme 16.**
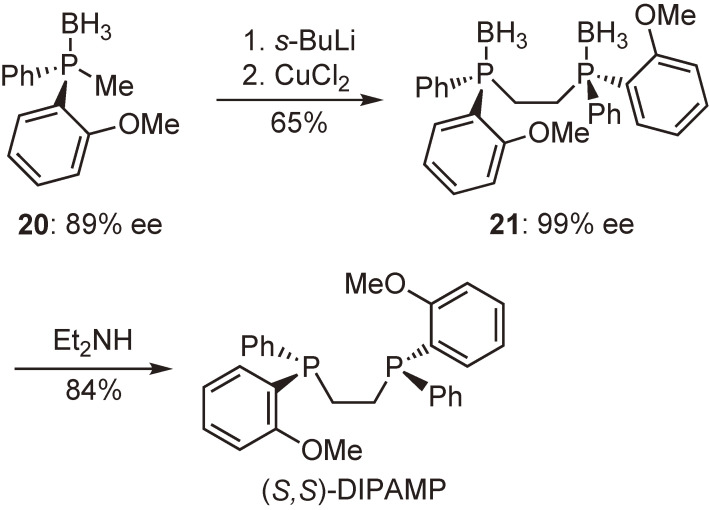
Synthesis of (*S*,*S*)-DIPAMP via phosphine–boranes.

Motivated by the results mentioned above, we tried to synthesize bidentate P-chiral phosphine ligands using phosphine–boranes as the intermediates. Our initial attempt commenced with the preparation of DIPAMP, as shown in Scheme 16. Compound **20** with 89% ee was successively reacted with *s*-BuLi and CuCl_2_ to give compound **21** with 99% ee after removal of the concomitantly formed meso-isomer. The subsequent reaction of **21** with diethylamine furnished enantiopure (*S*,*S*)-DIPAMP, demonstrating the potential utility of this phosphine–borane methodology.^39)^

**Scheme 17.**
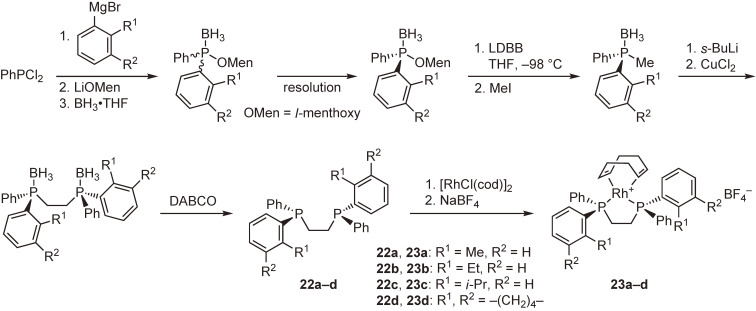
Synthesis of enantiopure (*S*,*S*)-1,2-bis[(*o*-alkylphenyl)phenylphosphino]ethanes and their rhodium complexes.

**Scheme 18.**
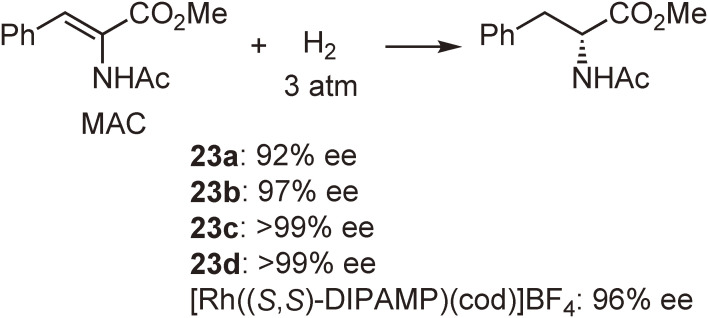
Asymmetric hydrogenation of methyl (*Z*)-α-acetamidocinnamate (MAC) catalyzed by **23a**–**d** and [Rh((*S*,*S*)-DIPAMP)(cod)]BF_4_.

In a similar manner, new P-chiral phosphine ligands **22a**–**d** and their rhodium complexes **23a**–**d** were prepared from dichlorophenylphosphine (Scheme 17), and their enantioinduction abilities were evaluated in the hydrogenation of a typical probing substrate, methyl (*Z*)-α-acetamidocinnamate (MAC). The hydrogenation results are shown in Scheme 18 together with that obtained by the use of a Rh-DIPAMP complex.

It is noted that complex **23b** bearing an *o*-ethylphenyl group provided remarkably high enantioselectivity (97%) comparable to that (96%) of [Rh((*S*,*S*)-DIPAMP)(cod)]BF_4_. Complexes **23c** and **23d** with larger *ortho*-alkyl substituents afforded almost perfect enantioselectivities (>99%), exceeding that of DIPAMP after 20 years.^56,57)^ Another significant fact is that the sense of the enantioselectivity of the newly synthesized ligands with (*S*,*S*)-configuration is the same as that of (*S*,*S*)-DIPAMP in the Rh-catalyzed asymmetric hydrogenation. These results clearly indicate that the coordinative interaction between methoxy oxygen atom and rhodium atom in the Rh-DIPAMP complex is not significant in the stereoregulation and the enantioselection is determined by the steric effects of the ligands.

### 3.2 Synthesis of electron-rich P-chiral phosphine ligands and their enantioinduction ability.

Although the above-mentioned ligands **22c** and **22d** exhibited higher enantioselectivity than DIPAMP, their molecular structures closely resembled DIPAMP, and hence we next tasked ourselves with the development of structurally new P-chiral phosphine ligands. In 1990–1991, Burk and coworkers reported that enantiopure 1,2-bis(*trans*-2,5-dialkylphospholano)ethanes (BPE) and 1,2-(*trans*-2,5-dialkylphospholano)benzenes (DuPhos), both of which are electron-rich bisphosphine ligands, exhibited exceedingly high enantioinduction abilities and catalytic efficiencies in Rh- and Ru-catalyzed asymmetric hydrogenations.^58–61)^ Inspired by their outstanding achievements, we concentrated on the design and synthesis of new P-chiral phosphine ligands.

**Figure 3.**
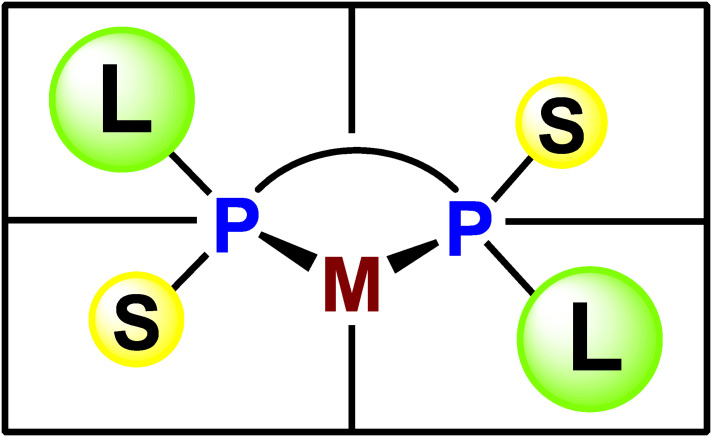
(Color online) Quadrant diagram of metal complexes of *C*_2_-symmetric P-chiral phosphine ligands.

**Scheme 19.**
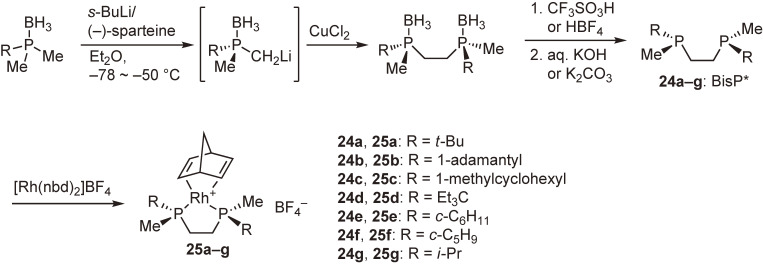
Synthesis of (*S*,*S*)-1,2-bis(alkylmethylphosphino)ethanes (BisP*) and their rhodium complexes.

Our ligand design concept was based on the quadrant diagram proposed by Knowles.^26)^ The newly designed ligands are *C*_2_-symmetric bisphosphines, in which a large alkyl group and a small alkyl group are bonded to each phosphorus atom, and the asymmetric environment around the center metal is quite clear (Fig. 3). This idea was immediately implemented in experiments of the synthesis of ethylene-bridged ligands, (*S*,*S*)-1,2-bis(alkylmethylphosphino)ethanes named BisP* (**24a**–**g**), possessing a tertiary or secondary alkyl group as the large group and a methyl group as the small group. The ligands were air-sensitive semi-solids or oils, and hence they were converted into air-stable rhodium complexes **25a**–**g** (Scheme 19).^62)^

**Figure 4.**
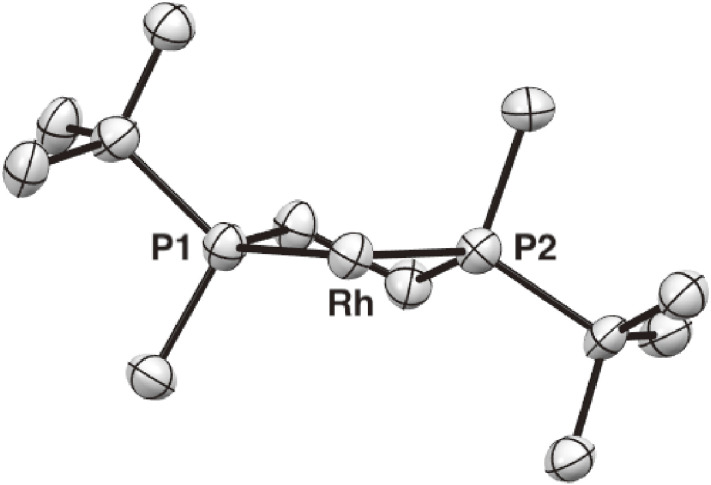
X-ray structure of [Rh((*S*,*S*)-*t*-Bu-BisP*)(nbd)]BF_4_ (**25a**). Coordinated norbornadiene, counterion, and hydrogen atoms are not shown for clarity.

The structure of complex **25a** having a *t*-butyl group as the large group was determined by single-crystal X-ray analysis. The ORTEP drawing shown in Fig. 4 clearly indicates the expected *C*_2_-symmetric environment, where the bulky *t*-butyl groups occupy the quasi-equatorial positions and the methyl groups are located at the quasi-axial positions to form a λ-chelate structure. This evidently defined asymmetric environment led us to anticipate the high utility of *t*-Bu-BisP* ligand in asymmetric catalysis.

**Scheme 20.**
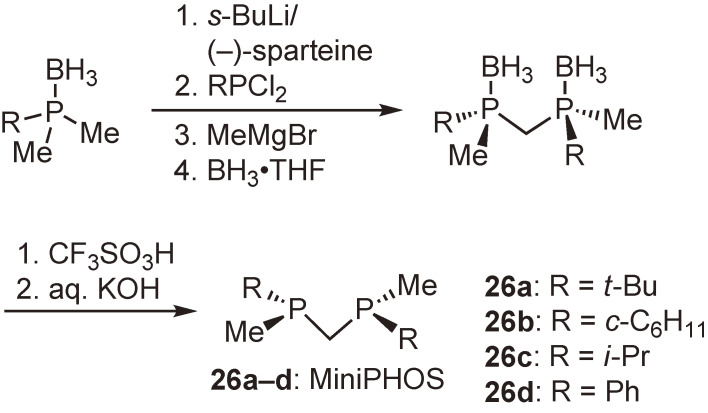
Synthesis of (*R*,*R*)-bis(alkylmethylphosphino)methanes (MiniPHOS).

**Figure 5.**
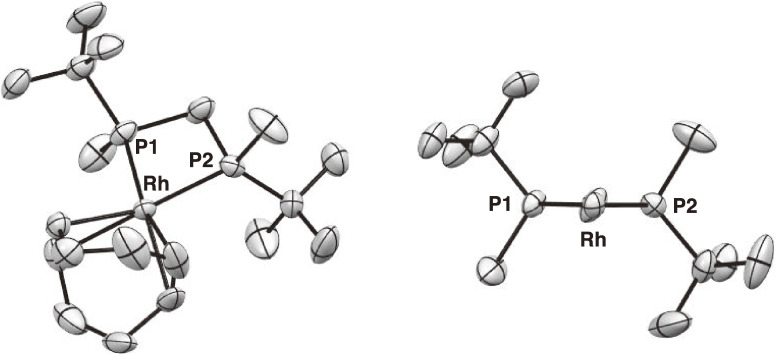
ORTEP drawing of [Rh((*R*,*R*)-*t*-Bu-MiniPHOS)(cod)]SbF_6_. The counter anion (SbF_6_^−^) and the hydrogen atoms are omitted for clarity. Left: perspective view; right: front view.

Subsequent to the above-mentioned work, we synthesized structurally simpler methylene-bridged bisphosphine ligands, (*R*,*R*)-bis(alkylmethylphosphino)methanes (MiniPHOS) (**26a**–**d**) (Scheme 20).^63)^ The complexation of *t*-Bu-MiniPHOS (**26a**) with Rh-diene complexes afforded two different four-membered Rh complexes depending on the diene ligand. Thus, the reaction of **26a** with norbornadiene complexes [Rh(nbd)_2_]X (X = BF_4_, PF_6_) provided bischelated complexes [Rh((*R*,*R*)-*t*-Bu-MiniPHOS)_2_]X,^63)^ whereas the reaction with a cyclooctadiene complex [Rh(cod)_2_]SbF_6_ afforded monochelated complex [Rh((*R*,*R*)-*t*-Bu-MiniPHOS)(cod)]SbF_6_.^64)^ Figure 5 shows an ORTEP drawing of the monochelated complex. It is noted that the four-membered chelate ring is almost entirely flat, the bulky *t*-butyl groups effectively shield the diagonal quadrants, and the two methyl groups locate on the other diagonal quadrants, constructing the expected asymmetric environment just as we designed. This *C*_2_-symmetric Rh-*t*-Bu-MiniPHOS complex is one of my favorite compounds because of its very simple structure.

The prepared Rh complexes of BisP* and MiniPHOS ligands were used in the asymmetric hydrogenation of α- and β-dehydroamino acid derivatives and enamides to evaluate their enantioinduction abilities. In most cases, the ligands possessing a *t*-butyl group exhibited very high enantioselectivities of up to 99.9%.^62,63,65–67)^ We were pleased to find that the two ligands, *t*-Bu-BisP* and *t*-Bu-MiniPHOS, were applicable to other representative catalytic asymmetric reactions, such as the Ir-catalyzed hydrogenation of imines and the Rh-catalyzed hydrosilylation of ketones.^68,69)^

**Figure 6.**
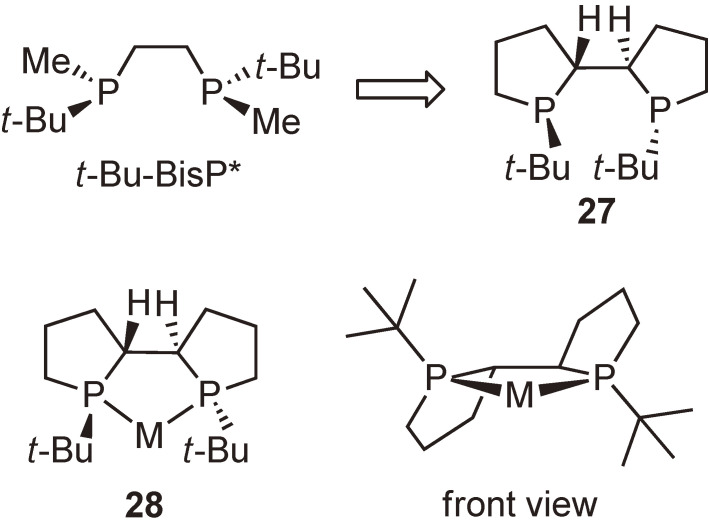
Newly designed P-chiral phosphine ligand **27** and its metal complex **28**.

Our next work was to develop more efficient chiral bisphosphine ligands. Day in and day out, we designed new P-chiral phosphine ligands using molecular models, finally designing ligand **27** consisting of two phospholane rings as our most promising candidate. The ligand was expected to form metal complex **28** having three fused five-membered rings, and the complex would be quite rigid to effectively block the two diagonal quadrants, eventually creating an ideal asymmetric environment around the metal center (Fig. 6).

**Scheme 21.**
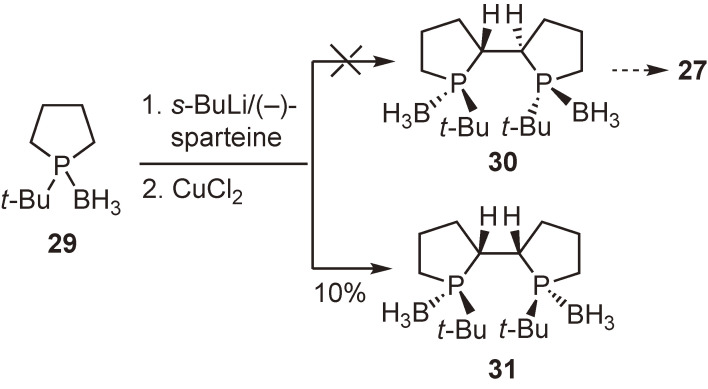
An attempt to synthesize ligand **27** from phosphine–borane **29**.

**Scheme 22.**
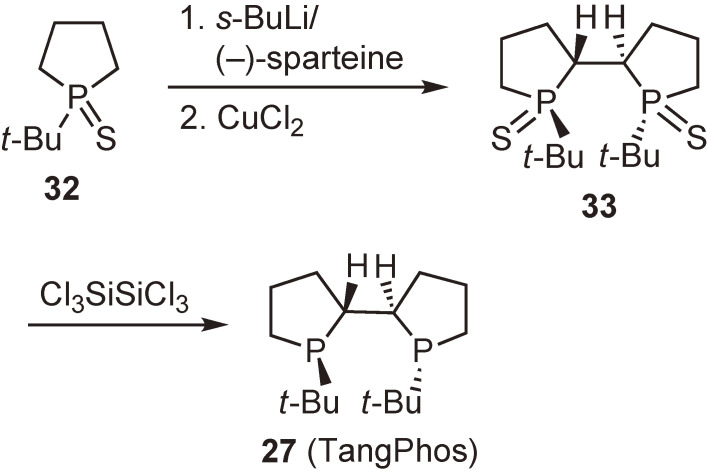
Synthesis of ligand **27** (TangPhos) by Zhang and Tang.

Initially, we presumed that ligand **27** could be readily synthesized from 1-*tert*-butylphospholane–borane **29** via *C*_2_-symmetric precursor **30**. However, the oxidative coupling proceeded sluggishly to form undesired meso-isomer **31** in 10% yield (Scheme 21).^70)^ To obtain the desired compound **30**, we further examined the coupling reaction under various conditions and at the same time sought other different synthetic routes; however, we were unable to isolate **30**. Meanwhile, I was astonished to find the paper by Tang and Zhang describing the synthesis and exceedingly high enantioinduction ability of ligand **27** (Scheme 22).^71)^ They used phosphine sulfide **32** as the starting material to obtain oxidative coupling product **33** as the major product. Actually, we had also carried out the experiments using the same phosphine sulfide **32**, but were unsuccessful in isolating the desired **33**. To this day, I very much regret not being more persistent in performing the experiments to achieve the first synthesis of ligand **27**.

**Figure 7.**
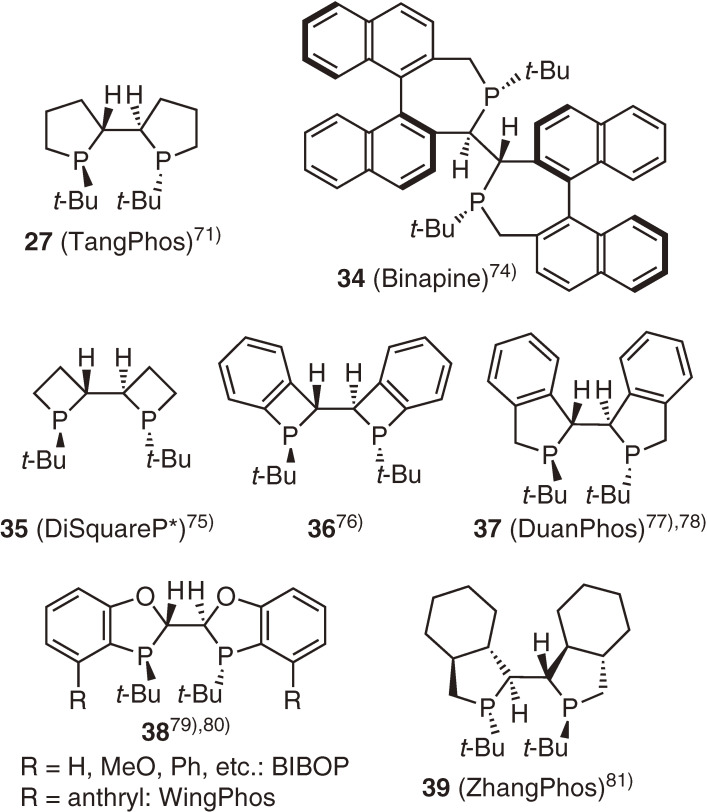
P-Chiral bisphosphacycle ligands with *t*-butyl groups.

The superior performance of TangPhos in not only Rh-catalyzed asymmetric hydrogenation reactions but also many other catalytic asymmetric reactions was demonstrated through extensive investigations by Zhang and Tang.^71–73)^ Following to TangPhos, analogous P-chiral bisphosphacycle ligands (**34**–**39**) bearing *t*-butyl groups at the phosphorus atom were reported (Fig. 7).^74–81)^ We also prepared ligands **35** and **36** consisting of more rigid four-membered phosphacycles, expecting that they would exhibit high enantioselectivity.^75,76)^ Among these ligands, Binapine (**34**),^74)^ DuanPhos (**37**),^77,78)^ BIBOP (**38**),^79,80)^ and WingPhos (**38**)^79,80)^ are widely used in both academia and industry for the production of many useful optically active compounds.^82)^ I am amazed at the powerful research activities of Professors Zhang and Tang and their coworkers. At the same time, I am delighted that these remarkable developments originated from the discovery of the *t*-Bu-BisP* ligand.

### 3.3 Synthesis of air-stable P-chiral phosphine ligands.

#### 3.3.1 2,3-Bis(tert-butylmethylphosphino)quinoxaline (QuinoxP*).

**Scheme 23.**
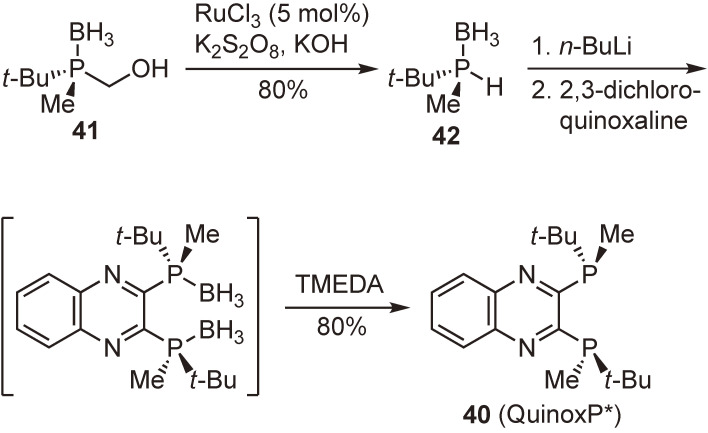
Synthesis of QuinoxP*.

Enantiopure 1,2-bis(*tert*-butylmethylphosphino)ethane (*t*-Bu-BisP*), a representative P-chiral phosphine ligand developed in our laboratory, exhibits very high enantioselectivities in several catalytic asymmetric reactions. However, this ligand is an extremely air-sensitive semi-solid, and this property has hampered its widespread application in catalytic asymmetric synthesis. In this context, I pursued a new ligand that is not an oily material but an air-stable crystalline solid to exhibit excellent enantioinduction ability similar to *t*-Bu-BisP*. Newly designed ligand 2,3-bis(*tert*-butylmethylphosphino)quinoxaline (**40**) (QuinoxP*) possessing the quinoxaline backbone was prepared from enantiopure (*R*)-*tert*-butyl(hydroxymethyl)methylphosphine–borane (**41**) (Scheme 23).^83)^ Compound **41** was transformed by a ruthenium-catalyzed stereospecific oxidative one-carbon degradation into (*S*)-*tert*-butylmethylphosphine–borane (**42**),^84)^ which was deprotonated with *n*-BuLi and allowed to react with 2,3-dichloroquinoxaline, followed by treatment with TMEDA to furnish the desired ligand **40** in an enantiopure form. We were overjoyed to obtain the ligand as an air-stable crystalline solid and to confirm its high performance in representative transition-metal-catalyzed asymmetric reactions.^83,85)^

**Figure 8.**
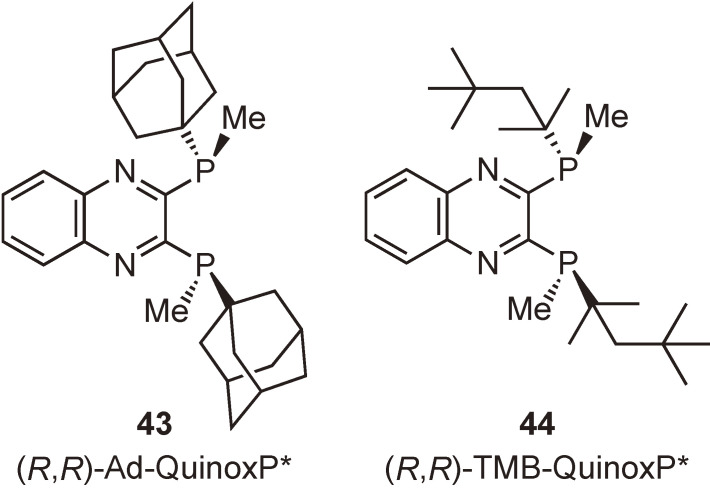
(*R*,*R*)-Ad-QuinoxP* and (*R*,*R*)-TMB-QuinoxP*.

In a similar manner, we prepared analogous *C*_2_-symmetric ligands, (*R*,*R*)-2,3-bis((1′-adamantyl)methylphosphino)quinoxaline ((*R*,*R*)-Ad-QuinoxP*) (**43**) and (*R*,*R*)-2,3-bis((1′,1′,3′,3′-tetramethylbutyl)methylphosphino)quinoxaline ((*R*,*R*)-TMB-QuinoxP*) (**44**) (Fig. 8). The former ligand, unfortunately, did not crystallize, whereas the latter one was obtained as orange plates. Both ligands showed excellent performance as chiral ligands in a few representative catalytic asymmetric reactions.^64,86)^

#### 3.3.2 1,2-Bis(tert-butylmethylphosphino)benzene (BenzP*) and related ligands.

**Scheme 24.**
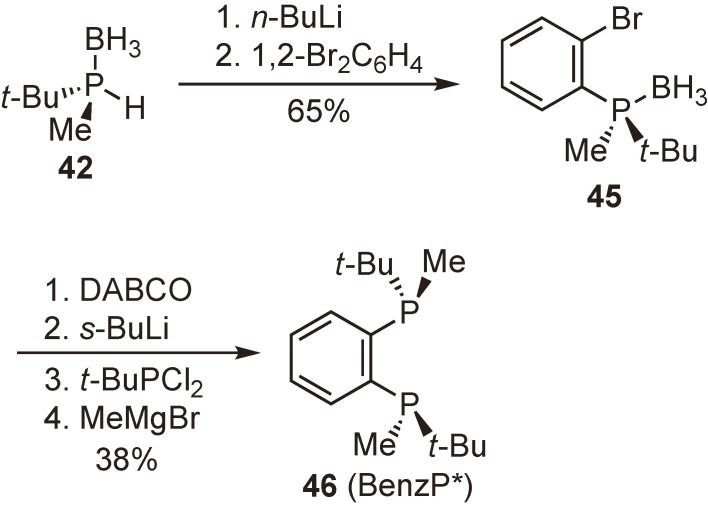
Synthesis of BenzP*.

For years, we were extremely interested in the synthesis of 1,2-bis(*tert*-butylmethylphosphino)benzene (BenzP*), an *ortho*-phenylene-bridged P-chiral bisphosphine ligand. Although the ligand has a simple molecular structure, our attempts to synthesize it took approximately 10 years. Finally, in 2010, we found a method that enabled gram-scale synthesis.^87–89)^ We owe our success to our private communication with Professor Sylvain Jugé of the University of Bourgogne. When I visited Professor Jugé’s laboratory, he kindly taught me the reaction of lithiated secondary phosphine–boranes with *o*-dibromobenzene to produce (2-bromophenyl)(dialkyl)phosphine–boranes.^90)^ By applying his protocol to the reaction of optically pure **42** with 1,2-dibromobenzene, we were able to obtain enantiopure **45** in good yield (Scheme 24). The conversion of **45** into BenzP* (**46**) in four steps was accomplished in one pot.^89)^ During the reaction sequence, the introduction of another *t*-butylmethylphosphino group at the *ortho*-position failed to proceed stereoselectively, resulting in the formation of a considerably large amount of the undesired meso-isomer as the co-product. Fortunately, however, only the desired (*R*,*R*)-BenzP* (**46**) was isolated as a colorless crystalline solid from the reaction mixture. It was also fortunate that the enantiopure BenzP* was considerably air-stable, in contrast to the mixture of its stereoisomers, which was reported to be an air-sensitive oil.^91)^ This air-stable property of the ligand in conjunction with its high enantioinduction ability makes it potentially useful in various catalytic asymmetric syntheses.

### 3.4 Synthesis of P-chiral bisphosphines via enantiopure tert-butylmethylphosphine–borane.

As has been described above, enantiopure *tert*-butylmethylphosphine–borane (**42**) plays a key role in the synthesis of QuinoxP* and BenzP*. I find this structurally simple secondary phosphine–borane very attractive, because it consists of a stereogenic phosphorus atom, a boranato group, a hydrogen atom, a methyl group, and a *t*-butyl group, all of which play respective roles in ligand synthesis or in the construction of an effective asymmetric environment. Whereas this compound was initially prepared on a laboratory scale as described in Schemes 9 and 23, its enantiomers are currently manufactured on a large scale through the optical resolution of the racemate at Nippon Chemical Industrial Co., Ltd.

**Figure 9.**
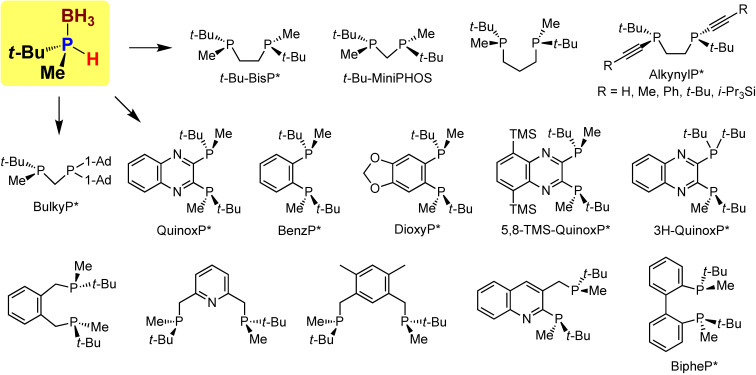
(Color online) P-Chiral phosphine ligands prepared from enantiopure *tert*-butylmethylphosphine–borane.

With both enantiomers in hand, we prepared various P-chiral phosphine ligands by the reactions with electrophiles, followed by the removal of the boranato groups. The ligands prepared by this method are illustrated in Fig. 9. It should be noted that both enantiomers of *t*-Bu-BisP* could be conveniently prepared from 1,2-dichloroethane.^64)^ Among these ligands, AlkynylP*,^92)^ BulkyP*,^93)^ 5,8-TMS-QuinoxP*,^94)^ and 3H-QuinoxP*^95–97)^ have shown unique and remarkably high enantioselectivities in some transition-metal-catalyzed asymmetric transformations.

## 4 Use of QuinoxP* and related air-stable phosphine ligands in transition-metal-catalyzed asymmetric reactions

Since the commercialization of QuinoxP* and BenzP* by reagent dealers, these ligands have been widely used in both academia and industry for the development of new transition-metal-catalyzed asymmetric reactions as well as for the synthesis of the desired optically active compounds. The successful results obtained by the use of these ligands are many, and hence, only representative examples are described herein.

### 4.1 Asymmetric hydrogenation.

**Scheme 25.**
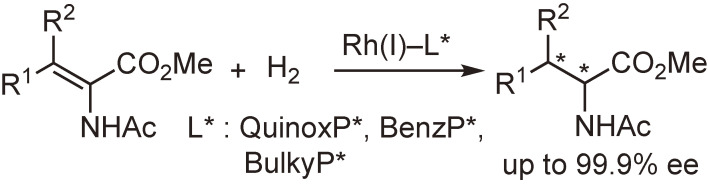
Rh(I)-catalyzed asymmetric hydrogenation of α-dehydroamino acid derivatives.

**Scheme 26.**
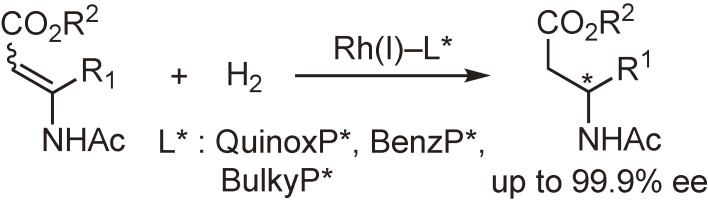
Rh(I)-catalyzed asymmetric hydrogenation of β-dehydroamino acid derivatives.

The rhodium-catalyzed asymmetric hydrogenation of functionalized alkenes is one of the most important catalytic asymmetric syntheses.^98,99)^ Even though plentiful examples have been reported, the further development of the method by using new ligands is still a popular research subject. We have examined the asymmetric hydrogenation of a variety of functionalized alkenes by the use of QuinoxP* and analogous ligands and found that the reaction resulted in excellent enantioselectivities and had a broad substrate scope. The hydrogenation reactions of α- or β-dehydroamino acid derivatives are shown in Schemes 25 and 26 as typical examples.^85,93)^ It is worth noting that the ligands could be employed in the hydrogenation of not only (*E*)- but also (*Z*)-β-dehydroamino acid derivatives (Scheme 26). These results indicate that the ligands, particularly QuinoxP*, are useful for the production of chiral ingredients containing an amino acid or amine moiety.^85)^

**Scheme 27.**
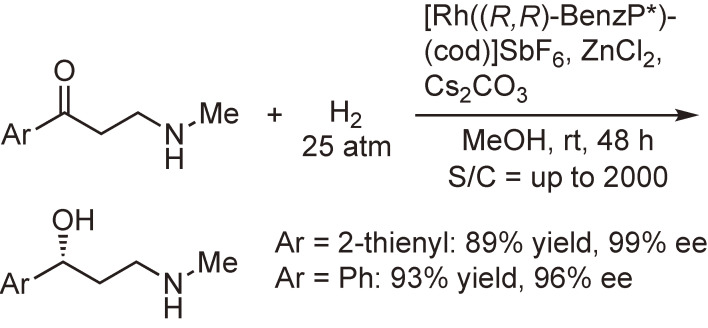
Rh(I)-catalyzed asymmetric hydrogenation of β-secondary-amino ketones.

High enantioselectivities have also been observed in the asymmetric hydrogenation of ketones using QuinoxP* or BenzP*. For example, the asymmetric hydrogenation of β-secondary-amino ketones by a Rh-BenzP* catalyst was significantly promoted by ZnCl_2_ to afford the corresponding hydrogenation products with excellent enantiomeric excesses in high yields (Scheme 27).^100)^ This procedure is potentially useful for the production of synthetic intermediates of (*S*)-duloxetine, (*R*)-fluoxetine, and (*R*)-atomoxetine, which are used as antidepressant drugs.

**Scheme 28.**
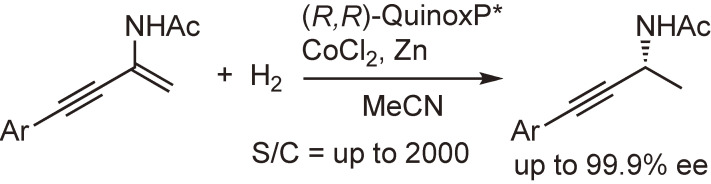
Cobalt-catalyzed asymmetric hydrogenation of conjugated enynes.

Recently, the use of non-precious metals, such as cobalt, nickel, and iron, in asymmetric hydrogenation has attracted increasing attention as an economical and environmentally friendly methodology. One reaction example using a cobalt-QuinoxP* catalyst is shown in Scheme 28.^101)^ In this reaction, the alkene moiety was hydrogenated with very high enantioselectivity while keeping the alkyne intact.

**Scheme 29.**
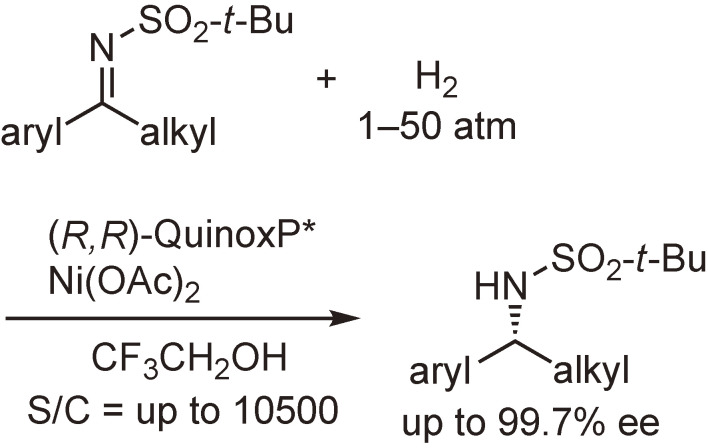
Ni-catalyzed asymmetric hydrogenation of *N*-sulfonyl imines.

Another example is the nickel-catalyzed asymmetric hydrogenation (Scheme 29).^102)^ The use of a much lower catalyst loading (0.0095 mol%, S/C = 10500) has resulted in the highest catalytic activity for the Ni-catalyzed asymmetric hydrogenation reactions reported to date.

### 4.2 Carbon–carbon and carbon–heteroatom bond-forming reactions.

**Scheme 30.**
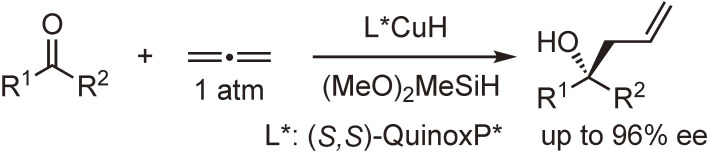
Enantioselective allylation of ketones with allene.

QuinoxP* and BenzP* have also been used in metal-catalyzed asymmetric carbon–carbon and carbon–heteroatom bond-forming reactions. Scheme 30 shows an example of a carbon–carbon bond-forming reaction reported by Buchwald and coworkers.^103)^ This allylation reaction of ketones with allene, an underutilized hydrocarbon feedstock, proceeds without specialized equipment or pressurization to give allylation products with high enantiomeric excesses.

**Scheme 31.**
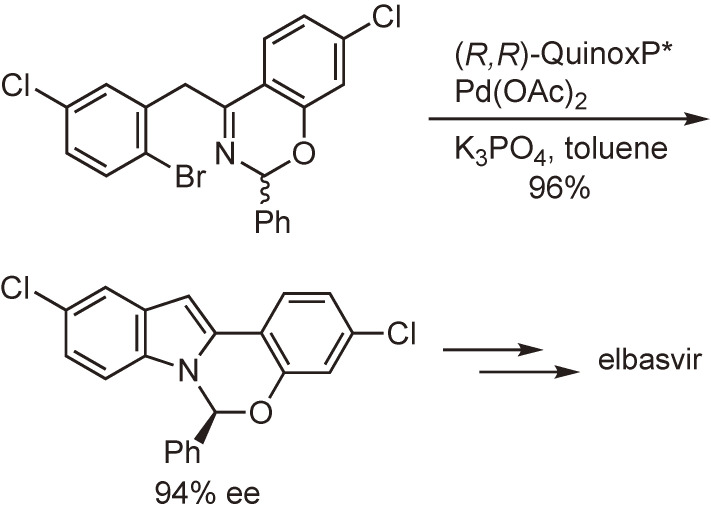
Synthesis of optically active hemiaminal ether via Pd-catalyzed C–N coupling.

Some air-stable P-chiral phosphine ligands have found use in the enantioselective synthesis of optically active pharmaceutical ingredients. Scheme 31 shows an example reaction that was established by the research group of Merck & Co., Inc. for the production of the HCV drug candidate elbasvir.^104)^ It should be noted that the C–N coupling reaction with the formation of the optically active hemiaminal ether is catalyzed by the Pd(0)–QuinoxP* mono-oxide complex produced by the reaction of Pd(OAc)_2_ with QuinoxP*.

**Scheme 32.**
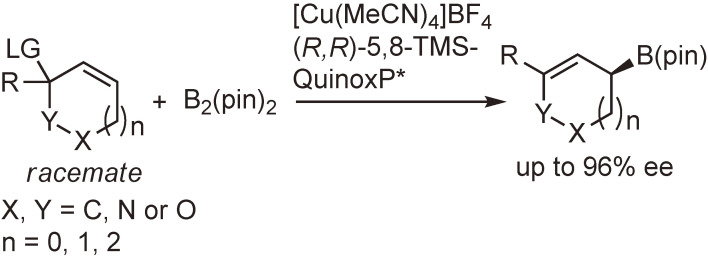
Direct enantio-convergent transformation of racemic substrates catalyzed by Cu(I)–(*R*,*R*)-5,8-TMS-QuinoxP* complex.

In 2010, Ito *et al.* disclosed a direct enantio-convergent transformation of racemic substrates without racemization or symmetrization by the use of Cu(I)–QuinoxP* complex as the chiral catalyst.^105)^ By employing the modified ligand (*R*,*R*)-5,8-bis(trimethylsilyl)quinoxaline ((*R*,*R*)-5,8-TMS-QuinoxP*), the substrate scope was significantly expanded from five-membered compounds to six- and seven-membered ones (Scheme 32).^94)^

## 5 Mechanistic study of rhodium-catalyzed asymmetric hydrogenation of functionalized alkenes: A new approach for predicting the sense of enantioselectivity

As has been described in Section 3.2, we found that the asymmetric hydrogenation of α-dehydroamino acid derivatives with rhodium complex [Rh((*S*,*S*)-*t*-Bu-BisP*)(nbd)]BF_4_ (**25a**) provided (*R*)-configuration products with up to 99.9% ee.^62)^ With the results in hand, we at first tried to explain the stereochemical outcome with respect to the hitherto reported empirical rule regarding the correlation between the catalyst δ or λ conformer and the absolute configuration of the products.^26,106–108)^ The rule predicts that the catalyst with δ-conformation provides (*R*)-enantiomer products and the catalyst with λ-conformation furnishes (*S*)-enantiomers. Therefore, according to the rule, our catalyst **25a** with λ-conformation should provide (*S*)-configuration products. However, it turned out that the use of **25a** afforded (*R*)-configuration products, which are the opposite enantiomers of those predicted by the empirical rule. On the other hand, in the case of the (*R*,*R*)-*t*-Bu-MiniPHOS (**26a**)–Rh complex, the δ,λ-empirical rule was not applicable to the entirely flat four-membered chelate cycle with no distinct quasi-axial and equatorial groups, although the complex afforded (*R*)-configuration products with excellent enantiomeric excesses. Another attempted explanation was made by applying the Halpern–Brown mechanism, which is also known as the unsaturated mechanism, the major/minor concept, or the anti-lock-and-key mechanism.^109–117)^ However, not only the origin and the sense of the enantioselection but also the observed very high enantioselectivities could not be reasonably explained by the mechanism. These discrepancies are the main reasons why we initiated our mechanistic studies of the rhodium-catalyzed asymmetric hydrogenation.

To detect the reaction intermediates and to examine their reactivities, we began our multinuclear NMR study with the use of complex **25a**. Fortunately, complex **25a** was sufficiently soluble in deuteriomethanol even at a very low temperature, and the spectral data obtained could be analyzed without difficulty owing to its simple molecular structure.

**Scheme 33.**
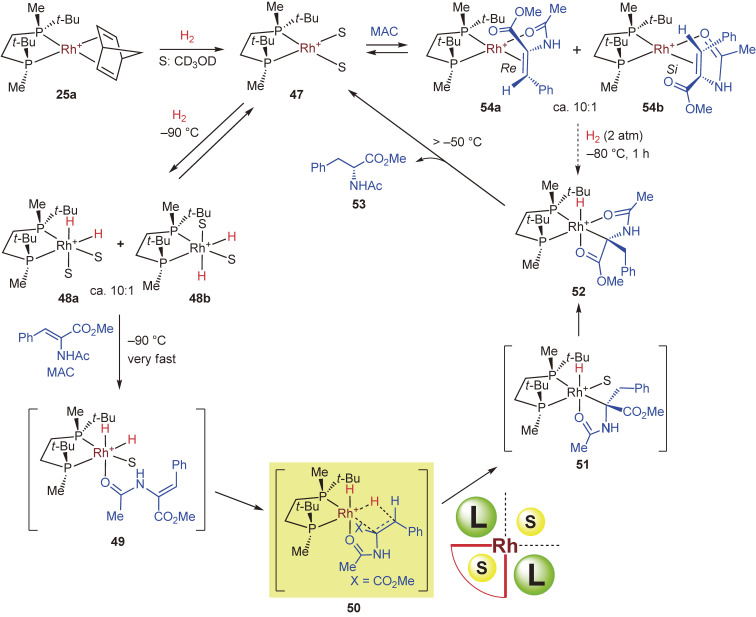
Plausible catalytic cycle for the asymmetric hydrogenation of MAC with [Rh((*S*,*S*)-*t*-Bu-BisP*)(nbd)]BF_4_.

Our experimental findings and a plausible enantioselection mechanism including the catalytic cycle are shown in Scheme 33.^118)^ One of the important findings is that solvate complex **47** generated from catalyst precursor **25a** reacted reversibly with H_2_ at a low temperature to give diastereomeric dihydride intermediates **48a** and **48b** in approximately 10:1 ratio; these are the first observable Rh dihydrides with a bisphosphine ligand. These dihydrides reacted with probing substrate MAC very rapidly at −90 ℃ to give monohydride complex **52** detected by NMR measurement. The reaction was considered to proceed through **49**, **50**, and **51**. Thus, the amide oxygen atom of MAC coordinated to the rhodium atom at the trans to the Rh–H bond to form complex **49**, and subsequently, the carbon–carbon double bond moved to the Rh–H bond, leading to the transition state **50**. The migratory insertion of the alkene into the Rh–H bond afforded monohydride complex **51**, which in turn isomerized into a more stable monohydride **52**. At temperatures above −50 ℃, **52** underwent reductive elimination to afford hydrogenation product **53** with 99% ee and solvate complex **47**.

On the other hand, solvate complex **47** reacted with MAC to form Rh-alkene complexes **54a** and **54b** in the ratio of approximately 10:1. These diastereomeric complexes were allowed to react with H_2_ (2 atm) at −80 ℃ for 1 h to give monohydride **52**, which was detected by NMR measurement along with the signals of solvate complex **47** and dihydrides **48a** and **48b**. At the temperatures higher than −50 ℃, it was converted into the hydrogenation product **53** with 97% ee (*R*). The absolute configuration of the product corresponded to that of *si*-coordinated minor complex **54b**, and this hydrogenation followed seemingly the Halpern–Brown mechanism. However, this transformation proceeded at a relatively higher temperature and required a longer reaction time than the reaction of the dihydride complexes **48a** and **48b** with MAC. Therefore, it is reasonable to consider that the alkene complexes **54a** and **54b** exist in a resting state and are not directly subjected to hydrogenation; they reversibly dissociate into the solvate complex **47** and substrate MAC and eventually furnished **52** via the dihydride pathway.

We consider that the enantioselection would be determined at the association step to form hexacoordinated Rh(III) dihydride complex **50**. Among the eight possible stereoisomers of the transition state structure, only **50** as the most favorable transition state satisfies the following steric and electronic requirements:

1. A chelate ring is formed, avoiding steric repulsion with the bulky *t*-butyl group of the ligand.
2. The carbon–carbon double bond is parallel to the Rh–H bond trans to the Rh–P bond.
3. The α-carbon of the ester binds to the Rh atom during the migratory insertion.

The origin of the very high enantioselectivity is responsible for the multiple factors for lowering the transition state energy. This enantioselection mechanism is analogous to that of enzyme reactions, even though the catalyst is not a macromolecule similar to an enzyme. It is also worth mentioning that the relationship between the catalyst structure and the product chirality can be reasonably explained by considering transition state structure **50**.

**Scheme 34.**
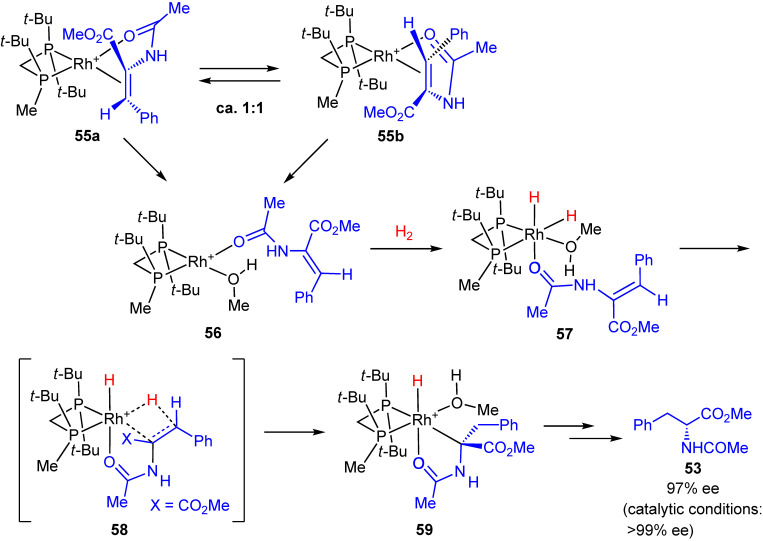
(Color online) Reaction of [Rh((*R*)-TCFP)]–MAC complexes with H_2_ leading to the hydrogenation product.

To confirm the dihydride mechanism, we studied the reactivities of tetracoordinated Rh(I)-alkene chelating complexes toward H_2_ using other electron-rich P-chiral phosphine ligands (*R*,*R*)-*t*-Bu-MiniPHOS, (*R*)-(*tert*-butylmethylphosphino)(di-*tert*-butylphosphino)methane (Trichikenfootphos: TCFP), (*R*,*R*)-BenzP*, *etc.* Scheme 34 shows an example of the studies based on low-temperature NMR measurements and DFT computations.^119)^ In this case, TCFP–Rh solvate complex reacted with MAC to provide alkene complexes **55a** and **55b** in approximately 1:1 ratio, and no distinct major/minor concentrations were observed. A significant finding is that both the *re*- and *si*-coordinated alkene complexes **55a** and **55b** reacted with H_2_ at −78 ℃ to give the same (*R*)-enantiomer product in 97% ee. The NMR monitoring and extensive DFT computations led us to conclude that the hydrogenation of **55a** and **55b** does not occur directly, but is preceded by the dissociation of the double bond to result in the more reactive species **56**. Semi-dissociated species **56** reacts with H_2_ to give non-chelating Rh(III)-dihydride complex **57**, which undergoes migratory insertion via transition state **58** to generate monohydride **59**. Finally, **59** is converted into product **53** via reductive elimination. It is noted that transition state **58** closely resembles **50** and the observed very high enantioselectivities would be responsible for the above-described steric and electronic effects.

In addition to the example mentioned above, the mechanisms of the Rh-catalyzed asymmetric hydrogenation of many functionalized alkenes, such as β-dehydroamino acid derivatives, enamides, and α,β-unsaturated phosphonates, were studied using not only (*S*,*S*)-*t*-Bu-BisP* but also other several P-chiral bisphosphine ligands. In all cases, the resulting high enantioselectivity and stereochemical outcome (*R* or *S*) were reasonably explained by considering the dihydride pathway. In addition, the absolute configuration of the product could be predicted by considering the transition structure in the association and migratory insertion step. Furthermore, the proposed catalytic cycle and the enantioselection mechanism would be crucial for the design of new chiral ligands and catalysts. More details are described in original papers^65–67,85,120–126)^ and reviews.^127–129)^

## 6 Conclusions

A new method for the synthesis of enantiopure P-chiral phosphine ligands has been established by utilizing the characteristic properties of phosphine–boranes. A rationally designed P-chiral phosphine ligand, *t*-Bu-BisP*, was synthesized in 1998, and its very high enantioinduction ability was demonstrated in the Rh-catalyzed asymmetric hydrogenation of functionalized alkenes. The discovery of the *t*-Bu-BisP* ligand ushered in a revival of P-chiral phosphine ligands in asymmetric catalysis, and many P-chiral ligands have been synthesized and successfully applied in a variety of transition-metal-catalyzed asymmetric reactions. In particular, TangPhos, DuanPhos, BIBOP, and QuinoxP* have found widespread use in both academia and industry owing to their exceedingly high enantioinduction ability and superior catalytic efficiency.

Structurally simple P-chiral phosphine ligands, such as BisP*, TCFP, and BenzP*, have been employed in the mechanistic studies of the Rh-catalyzed asymmetric hydrogenation of enamides and related substrates. It has been suggested that the hydrogenation proceeds through the dihydride pathway and the enantioselection is determined by the formation of the octahedral Rh(III) dihydride complex and the subsequent migratory insertion step. Furthermore, the absolute configuration of the products can be predicted by considering the multiple stereoregulating factors at the transition state.

Asymmetric catalysis is undoubtedly one of the most environmentally benign and economical methods for the production of enantiomerically pure or enriched high value-added compounds. I believe that P-chiral phosphine ligands along with many other backbone chirality ligands will play a vital role in the further development of the sophisticated and truly useful catalytic synthetic transformations.
